# Social Gaming to Decrease Loneliness in Older Adults: Recruitment Challenges and Attrition Analysis in a Digital Mixed Methods Feasibility Study

**DOI:** 10.2196/52640

**Published:** 2024-10-16

**Authors:** Bas D L Châtel, Jeroen H M Janssen, Geeske M E E Peeters, Rense Corten, Rob Tieben, Menno Deen, Elmy J M Hendriks, Marcel G M Olde Rikkert

**Affiliations:** 1 Department of Geriatric Medicine Radboud University Medical Center Nijmegen Netherlands; 2 Computational Science Lab University of Amsterdam Amsterdam Netherlands; 3 Radboudumc Alzheimer Center Radboud University Medical Center Nijmegen Netherlands; 4 Department of Sociology/Interuniversity Center for Social Science Theory and Methodology Utrecht University Utrecht Netherlands; 5 Games for Health Eindhoven Netherlands; 6 Super Menno Monster Utrecht Netherlands; 7 Department of Geriatric Medicine Donders Institute for Brain, Cognition and Behaviour Radboud University Medical Center Nijmegen Netherlands

**Keywords:** loneliness, digital health, serious gaming, older adults, recruitment, feasibility study

## Abstract

**Background:**

Digital mental health interventions could sustainably and scalably prevent and reduce loneliness in older adults. We designed an app containing 29 text-based games and a questionnaire-administering chatbot to stimulate intergenerational contact.

**Objective:**

This study aims to evaluate the feasibility of a social gaming app in reducing loneliness among older adults by evaluating recruitment strategies, data collection procedures, and gameplay activity.

**Methods:**

This mixed methods study recruited participants via newsletters, articles, and a social media campaign. We used semistructured interviews and descriptive analysis of questionnaire answers and game data to assess feasibility. Key measures included recruitment reach and efficiency, participant demographics, in-app activity, and app usability and engagement feedback.

**Results:**

The social media campaign reached 192,641 potential participants, resulting in 1363 game downloads. A total of 155 participants (aged 65 years and older: n=34, 21.9% and aged less than 65 years: n=121, 78.1%) provided informed consent, yielding a conversion rate of 0.08%. The recruitment campaign focusing on distanced playful interaction had a significantly (*P*<.001) higher click-through rate (1.98%) than a campaign focusing on research participation (click-through rate=0.51%; *P*<.001). The overall conversion rate from advertisement exposure to research participation was 0.08%. Participants had a mean age of 48 (SD 16) years. The 65 years and older group averaged 70 (SD 5) years, while the less 65 years group averaged 42 (SD 12) years. Additionally, 45.2% (57/126) reported at least moderate levels of loneliness at baseline. Of the initial 554 players, 91 (16.4%) remained active after the first week, and 32 (5.8%) remained active for more than 90 days. Active participants tended to interact with those within their own age group, as indicated by a Pearson correlation of r=0.31 between the ages of the message sender and receiver. Interviews with 12 (48%) participants (aged 65-79 years; female: n=12, 83%) revealed barriers such as excessive chatbot questions and a mismatch between the target group and app design focus. Questionnaire completion rates dropped from 46% at baseline to 10% at follow-up.

**Conclusions:**

These findings underscore the challenges of recruitment and retention for older adults in a fully digital social gaming intervention. Effective recruitment strategies and targeted app design are crucial for engagement. Based on these insights, future interventions should focus on simplified interfaces, clear guidance for gameplay, and addressing the specific needs and preferences of older adults, thereby enhancing the effectiveness of digital mental health interventions.

## Introduction

In the Netherlands, people younger than 55 years (20%) and 95 years (62%) experience moderate to severe loneliness [1] according to the De Jong Gierveld Loneliness Scale (DJG) [2,3]. Loneliness increases with age due to social network changes, availability of family members and friends, opportunities for transportation, and health problems [4-6]. Loneliness, in turn, exacerbates these health factors [7], thus creating a negative feedback loop.

There are 2 reasons for limited robust evidence for loneliness interventions: a lack of randomized, blinded trials [8-14] and the difficulty with recruitment. Lonely participants are hard to include in studies, as people typically do not self-identify as lonely or fear the stigma of loneliness [15].

Local interventions are often not scalable without demanding vast resources and are often not transferable to other contexts [16-20]. Digital mental health interventions (DMHIs) might increase efficiency and reach, standardize data gathering, and reduce the costs of care and workforce needed [21]. Such DMHIs have been successfully used in several psychotherapeutic, cognitive remediation, and telepsychiatric interventions and applied to healthy aging and disorders such as attention-deficit/hyperactivity disorder and dementia [22]. The use of DMHIs to reduce loneliness in older adults is scarce. During the COVID-19 pandemic, DMHIs were successfully used to reduce loneliness. However, these DMHIs were not easily scalable and did not focus on strengthening the social networks of an older population [16,23,24].

Applied gaming is a type of DMHI using the idea of “serious gaming” to achieve goals other than entertainment [22]. It adds playful design and game dynamics elements to an intervention to, for example, promote engagement, enhance intervention efficiency and efficacy, and possibly induce behavioral change [25]. Social gaming, a specific form of serious gaming, stimulates the creation of friendships and social interaction [26-29] and can mitigate feelings of loneliness [29]. However, game mechanics and personal factors such as stress levels may affect individual outcomes [30,31]. For example, 1 study found that individuals playing to increase social interaction may experience less loneliness, but people playing for diversion from daily life may feel lonelier [31]. As such, social games need to be designed and evaluated purposefully, as the effect of gaming on loneliness may vary across individuals.

This study evaluated a loneliness-targeting gaming intervention co-designed with game developers, design experts, and representatives from aging communities. Our research aim was to describe the recruitment and inclusion process and search for options for improved efficiency in games for health studies.

## Methods

### Ethical Considerations

The local ethics committee of the Radboud University Medical Center (2020-6645) reviewed the study and judged it not to fall within the remit of the Medical Research Involving Human Subjects Act. Therefore, the ethics committee approved the study based on the Dutch Code of Conduct for Health Research and Responsible Use, the Dutch Personal Data Protection Act, and the Medical Treatment Agreement Act. Participants provided informed consent in-app before data collection. No compensation was provided to participants, and data was securely stored in a database compliant with European laws (SO 9001, 27001 and NEN 7510), ensuring privacy and confidentiality according to the Dutch Personal Data Protection Act.

### Participants

The research aimed to streamline the recruitment and inclusion process and identify ways to enhance efficiency in health-related gaming studies. This study did not involve specific sample size calculations for the recruitment aim, although our original study focused on measuring social interaction through gaming. For details on the sample size calculation of the original study, see Multimedia Appendix 1 [32-34].

We recruited participants via newsletters, studies, workshops, project websites, and all project partners’ networks and social media channels (mainly through Facebook campaigns). Recruitment through the social media campaign lasted from August 2020 to February 2021 (ie, 6 months). The app remained available after the campaign ended. Recruitment efforts mainly targeted potential players aged 65 and older. However, younger players were also targeted to allow younger players to invite, assist, and play with older players. For example, we organized a gaming cocreation workshop with children and included one of the games they created in the app.

No exclusion criteria were applied to prevent in-game social network formation restrictions, allowing everyone to download the app. Participants were divided into 2 groups for analysis: older adults are those aged 65 years and older (“65+”) and younger adults are those younger than 65 years (“65–”) in accordance with the United Nations’ definition [35]. We chose 65+ as the cutoff age because this period is characterized by retirement (the average age at retirement is 65 years and 11 months [36]) and good health, often referred to as “the third age” [37]. This group is also considered well-suited for preventative measures against loneliness later in life [38].

After downloading the app, agreement to the terms of conditions was mandatory to use the app, which included sharing backend data (eg, message type and timestamp and game session information). Participants were then provided information, asked for informed consent, and administered questionnaires by a chatbot. Finally, Dutch-speaking participants 65 years or older could enroll in poststudy interviews assessing user experience regarding game and study design. For more information on participant demographics, see Multimedia Appendix 2.

### Playing Together App

#### Development Process

The PlayingTogether (“SamenSpelen”; Games for Health) app development used an iterative design process between the research team, the game developers, and the target group (Multimedia Appendix 3).

#### Game Description

PlayingTogether is a photography and text-based social gaming platform on iOS and Android consisting of 29 mini-games designed to elicit personal social interaction. These games are chat groups allowing synchronous and asynchronous interaction, themed around a specific set of rules. Examples include playing Hangman (“Galgje”) and exchanging photographs of objects starting with the last letter of the thing depicted in the previous photo (“Fotoslang”).

#### User Interface (UI)

There were 2 major UI versions during this study. The first version was available at launch containing a black-and-white appearance with line art icons as game avatars. The black and white color scheme allowed less distraction regarding design and game mechanics during iterative feedback rounds with target users, as gaining feedback on these aspects was the main goal in this initial phase.

The design closely resembled commonly used messenger apps to facilitate user-friendliness, as over 70% of Dutch people aged 65-75 years used messaging apps in 2019 [39]. However, we focused game design on compatibility with all age groups to make the app more appealing to younger family members and promote inter-generational gameplay. Figures S4A-S4C in Multimedia Appendix 4 show the first version’s home page, game repository, and conversational chatbot, respectively. Subsequently, according to a user voting campaign via Facebook, we introduced a new UI, as seen in Figures S4D-S4F in Multimedia Appendix 4. The updated design was developed to aim for a fun and playful feeling for all ages.

### Data Collection

We collected data through semistructured interviews and the app. The app collected data through a questionnaire administering chatbot, helping to boost completion rates as participants can stay in the app to answer questionnaires in a conversational format. In addition, it provides game data by storing backend information regarding gameplay.

#### Recruitment Data

The numbers of impressions and clicks were stored to monitor the reach versus the effectiveness of the recruitment campaign. Impressions refer to the number of people seeing the ad, while clicks refer to the number of people clicking on the link provided within the ad.

#### Questionnaires

The chatbot was preprogrammed to administer 5 questionnaires at baseline. Participants started by answering a Personal Information Questionnaire (PI) containing questions regarding, for example, marital status, work status, living situation, and education. Next, a mobility score was calculated using the Life Space Assessment [40], which has been translated into several languages with acceptable validity and reliability [41,42], and more recently translated, though not yet validated, for Dutch older adults [43]. This score ranges from 0=totally bed-bound to 120=independently traveled out of town daily [44]. Next, loneliness scores were measured at baseline and repeated after 1, 2, and 3 months as the primary outcome measure by DJG, which was developed and validated in the Netherlands [2,45] and translated to several different languages later on [46]. It consists of a 6-item scale for emotional loneliness and a 5-item scale for social loneliness, with the total loneliness score being categorized into 4 levels: 0-2=not lonely, 3-8=moderately lonely, 9-10=severely lonely, and 11=very severely lonely. The Older Persons and Informal Caregivers Survey, developed and validated in the Netherlands [47,48], provided information on demographics, morbidity, quality of life, functional limitations, emotional well-being, social functioning, and health services use [47]. Finally, the Network Domain Identification and Significance (NDIS) questionnaire, developed and validated in the Netherlands [32], provided information on participants’ social networks at baseline and end of participation. Participants aged 65+ had to answer all questionnaires, while younger participants only answered the PI and DJG questionnaires.

#### Game Data

Game data entails a session creation timestamp, a list of player IDs, session IDs, and game names. We stored message length, timestamp, sender ID, and type (text or photo) for every message sent within a session. Social network data is stored implicitly through message receivers and senders. Player IDs were linked with profile creation time at first login and, if given, their age.

#### Interviews

Participants who agreed to be contacted for a qualitative evaluation were asked to participate in a semistructured telephone interview in March 2021. All interviews were voice recorded and conducted by a female project member (EH) according to an interview guide (Multimedia Appendix 5) with primarily open-ended questions structured around the following main topics: barriers and facilitators regarding the app, games, and chatbot. Participants provided informed consent before their interviews, field notes were taken during the interviews, and the audio recordings were transcribed verbatim and anonymized afterward. Data saturation was reached after 12 interviews.

### Analysis

#### Assessment of App Download Funnel

We applied A/B testing to compare social media recruitment strategies where *A* focused on participation in research and *B* focused on distanced playful interaction. Each focus was divided into 3 different emphases. For focus *A*, the emphasis was on *A*_1_: reducing loneliness in older adults, *A*_2_: studying the effect of gaming on valuable interpersonal contact, and *A*_3_: the impact of gaming on well-being. Focus *B* involved the emphasis on *B*_1_: the need to play together due to the COVID-19 pandemic, *B*_2:_ the app facilitating more family time, and *B*_3_: encouraging play on special occasions often used for social interaction (eg, Christmas).

We used Facebook’s A/B testing functionality to analyze the effectiveness of these recruitment strategies. This method randomly advertised the 2 focuses and their underlying emphases while recording their total number of impressions and the number of resulting clicks. Then, the click-through rate (CTR), that is, clicks divided by impressions, and total conversion rate (CR), that is, the total number of app downloads divided by impressions, were calculated from these numbers to measure recruitment strategy effectiveness. Finally, a *z* test for independent proportions was used to calculate whether CTRs significantly differed between advertisement focus *A* and *B*.

To assess recruitment effectiveness outside social media channels, the chatbot also asked participants how they learned about the app or study: advertisement or paper, newsletter, acquaintance, project website, social media, or other.

#### Assessment of In-App Participant Funnel

We assessed the total number of active profiles at critical points in the participant journey to uncover bottlenecks in app usage. This entailed evaluating the number of people who gave consent to the terms and conditions, agreed to read and provide informed consent to the questionnaire chatbot, answered the questionnaires at different time points, and sent messages.

#### Analysis of In-Game Collected Data

Python (Python Software Foundation) was used for a descriptive analysis of sample characteristics, usage dynamics, and social network information. The degree distributions are described using the mean, minimum, maximum, and SD. Furthermore, the median is reported with the first and third quartiles. A preliminary descriptive analysis of app effectiveness was performed by assessing DJG scores over time. Furthermore, the Pearson correlation *r* is used on message data to relate the age of the message’s sender and receiver, providing an outcome between *r*=–1 and *r*=1. A value of 1 means that everyone only communicates with their own age group, –1 means that everyone avoids communicating with their own age group, and 0 implies that communication is random regarding age. When analyzing network structure, we refer to components, that is, islands of connected players in which no connections exist between players on other islands. Using these components could increase an individual’s social network naturally as it provides information to introduce the individual to previously unknown friends of friends. Therefore, nonfully connected components (ie, not everyone in the component knows one another) could be used for targeted introductions and network growth.

#### Analysis of Interviews

The interview transcripts were coded using ATLAS.ti (ATLAS.ti Scientific Software Development GmbH) and analyzed thematically to identify relevant and overarching themes [49]. Two researchers (JJ and EH) independently coded the interviews, discussing codes and differences afterward.

## Results

### Overview

Among the 155 participants who provided informed consent, the average age for all participants was 48 (SD 16) years. The “65+” group had an average age of 70 (SD 5) years, while the “65–” group had an average age of 42 (SD 12) years. Both groups exhibited characteristics of being predominantly healthy, highly educated, married, female, living independently with others, and having children (Multimedia Appendix 2). In total, 57 out of 126 (45.2%) participants who completed the DJG questionnaire at baseline reported at least moderate levels of loneliness. The subgroup of 13 out of 27 (48.1%) participants aged 65 and older, experienced moderate loneliness, while 8 out of those 27 (30%) reported severe loneliness. Twenty-five participants gave consent to be contacted by the researchers, of whom 12 (48%; female: n=12, 83%) consented to be interviewed.

### Participant Recruitment

#### Overview

**Figure 1:**
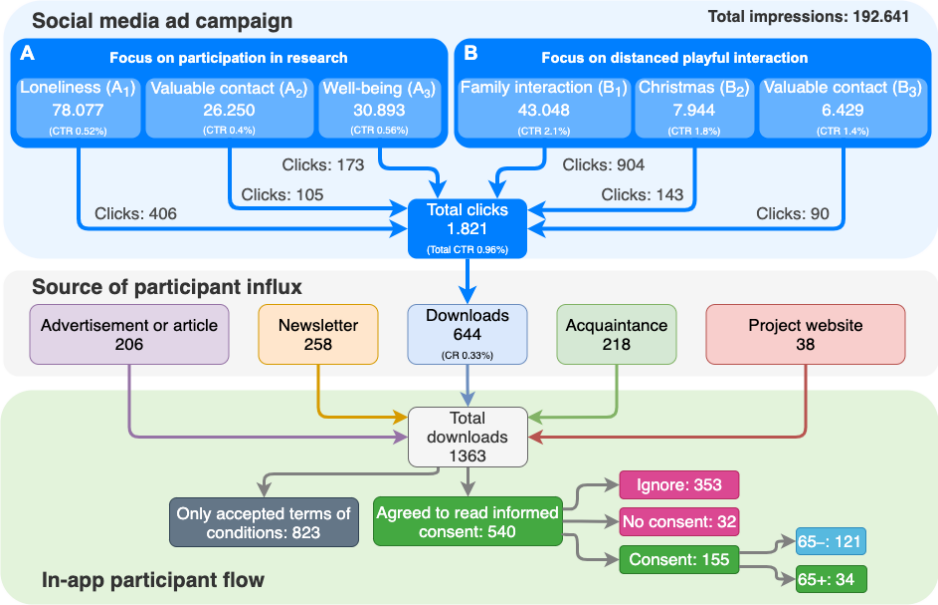
The flow of potential participants from ad exposure to informed consent. On the top level, the number of impressions is shown with the number of clicks and CTR per ad focus and emphasis. The middle level depicts the origin of how participants found the app. The bottom level illustrates in-app participant flow through different consent stages, ending with the number of participants 65 years or older (65+) and participants younger than 65 (65–). CTR: click-through rate.

Figure 1 depicts a higher CTR for focus (*P*<.001) on distanced playful interaction (CTR=1.98%) than focus *A* on research participation (CTR=0.51%). However, the reach of focus *A* was greater (70.2% of total impressions). Focus *B* generated more clicks, with 49.64% of the total number of clicks from the campaign emphasizing family interaction (*B*_1_: CTR=2.10%, number of impressions=22.35%). The total CR of the social media campaign was 0.33%.

Of the 1363 downloads, 823 participants only agreed to the mandatory terms and conditions, and 540 participants also agreed to receive research participation information, where the chatbot asked for informed consent. Of these 540 participants, 353 (65.3%) ignored the chatbot, 32 (5.9%) refused, and 155 (28.7%) gave informed consent. Among the 155 participants, 121 (78.1) were 65–, while 34 (21.9) were 65+. The total CR from advertisement exposure to research participation was 0.080% (ie, number of consent/impressions=155/192.641). As channels other than social media were also used, this total CR is an upper bound.

**Figure 2:**
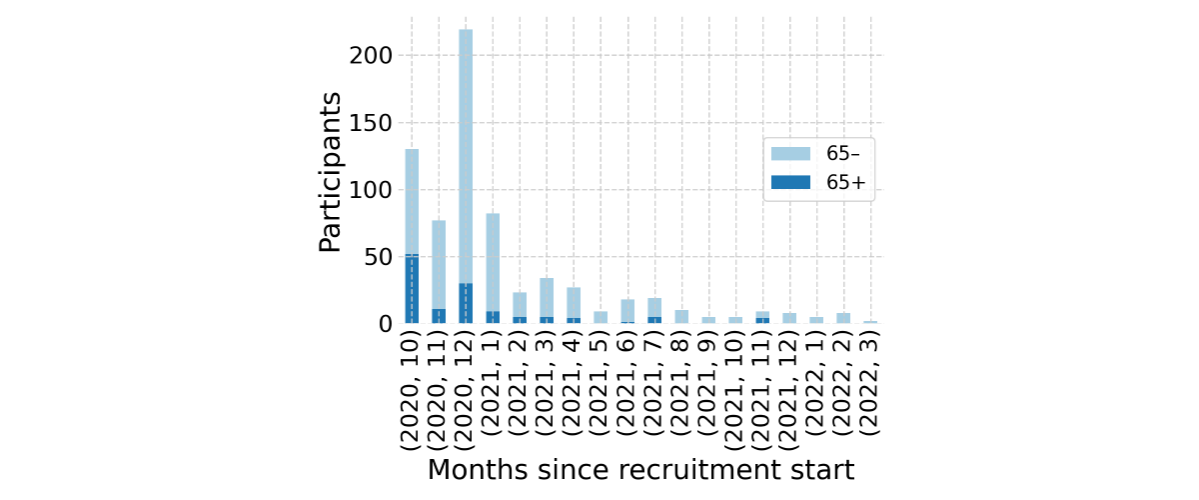
Participant influx over time per age group since the start of recruitment.

Regarding timing, Figure 2 shows an increase in participants at the app’s launch due to initial recruitment efforts. A participant inflow peak follows due to the recruitment campaign between December 18, 2020, and January 4, 2021, followed by a decreased growth, stagnating at around 10 new participants per month. This stagnation indicates the importance of active and continuous recruitment efforts.

#### Feedback Through Qualitative Interviews

Some interviewees indicated that they did not invite someone else because the invitation process was unclear. Furthermore, some indicated that the app was not enjoyable enough to invite someone. One of the participants said:

It was a barrier for me that I was going to involve others in something that annoyed myself.

Participant, female, 78

### Data Collection Procedures and Intervention Acceptability

#### Overview

**Figure 3:**
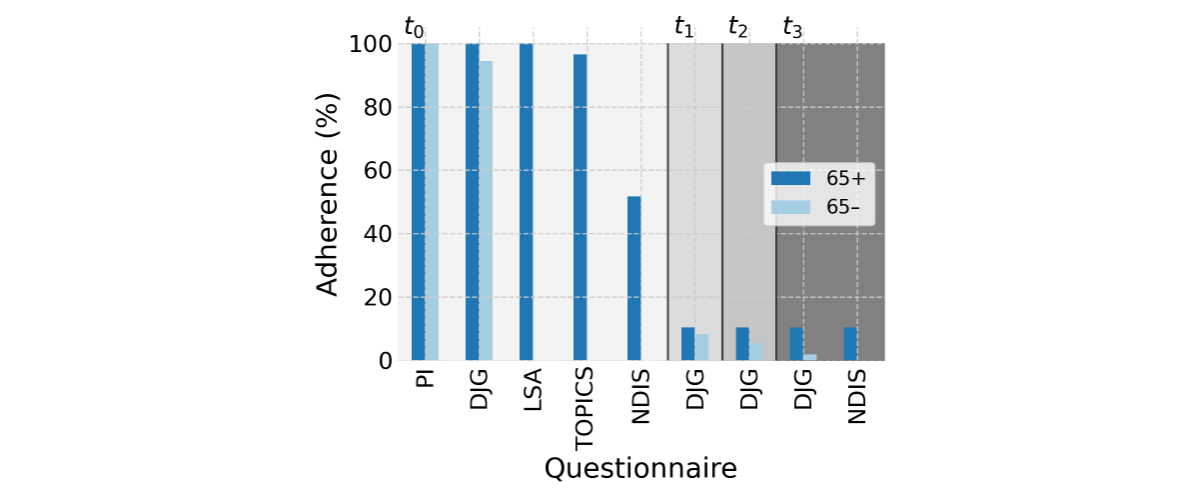
Questionnaire adherence compared to baseline (t0), with follow-up questionnaires each consecutive month. PI: Personal Information Questionnaire; DJG: De Jong-Gierveld Loneliness Scale; LSA: Life Space Assessment; TOPICS: The Older Persons and Informal Caregivers Survey; NDIS: Network Domain Identification and Significance.

Questionnaire adherence was relatively steady for PI (N=29, N=109), DJG, and the Life Space Assessment questionnaires at baseline. However, there was a drop from 28 to 15 participants (a 46% drop) who completed the NDIS questionnaire after completion of the Older Persons and Informal Caregivers Survey questionnaire (Figure 3). During the following months, questionnaire completion rates were further reduced to 3 participants (10% relative to baseline), after which the 65+ group’s completion rate remained stable while the 65– group continued to decline.

#### Feedback Through Qualitative Interviews

Most interviewees expressed that the chatbot presented information pleasantly and explained the study goal well. However, to some, the chatbot felt slightly artificial, childish, or communicated too quickly. In addition, for some questions, the goal was judged unclear, so interviewees did not feel motivated to answer these questions, as 1 participant illustrated:

I did not exactly understand what it was about. So was that questionnaire, with all those contacts, to see if I have contacts? Or to see if I have many contacts? Or was it about finding people with whom I wanted to play a game? That was not clear.

Participant, male, 77

Interviewees also indicated that the questionnaires contained too many questions, making the average questionnaire duration unpleasant. One of the participants indicated:

I was filling out questionnaires all the time, and I didn’t like that.

Participant, male, 77

When asked how often interviewees were willing to answer questions, they indicated that monthly would be fine, provided it was not too long. Additionally, the NDIS required participants to disclose information about others, which many interviewees did not appreciate.

### Participant Gameplay

#### Overview

**Figure 4:**
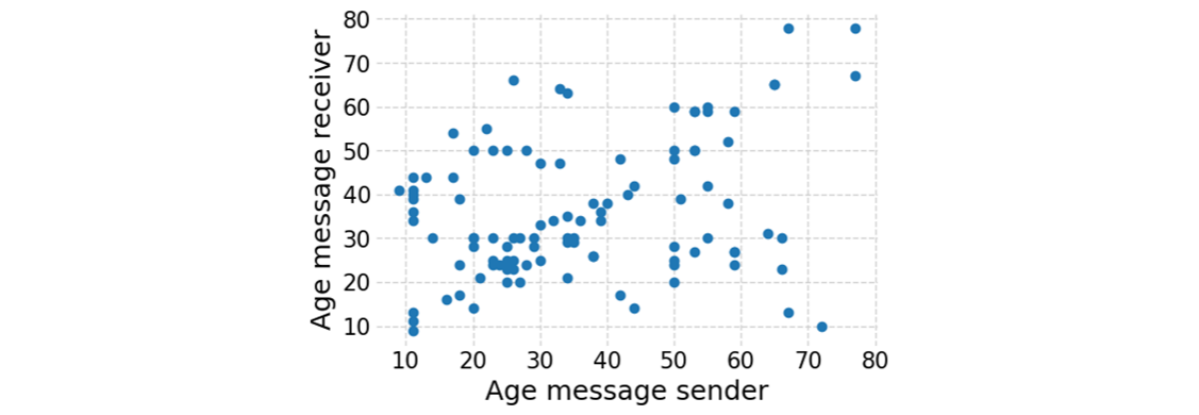
Age of message sender and receiver. Each dot represents a message from the sender (x-axis) to the receiver (y-axis), based on their respective age.

Out of the initial 554 participants, 91 (16.4%) remained active after the first week, and 32 out of the 554 (5.8%) participants had more than 90 days of activity. Furthermore, assessing design choices toward intergenerational play, we found participants tended to play with players in their age stratum (*r*=0.31; Figure 4 and Multimedia Appendix 6).

#### Feedback Through Qualitative Interviews

Interviewees mentioned that the games were amusing, intuitive, and clearly explained. The games were often considered somewhat slow, old-fashioned, and childish in terms of content and appearance. Some indicated that the games should be more challenging and usually stopped playing after a few times. One of the participants mentioned:

Yes, I find it a bit, simple. They are party games, and there is little challenge in it.

Participant, female, 66

The number of games was considered enough by some and too low by others. Furthermore, interviewees mentioned not seeing the added benefit of the games in this app over established digital games. Interviewees mentioned that the games are most suitable for playing with children rather than with other adults. However, the interviewees who invited someone else generally invited a good friend or partner of the same generation.

## Discussion

### Principal Findings

This study investigated the recruitment and inclusion process of a serious game app to reduce loneliness in the older population. Due to the high dropout during the recruitment and onboarding process, substantial overshooting of the intended sample size was required. Of the 193,361 potential participants reached through social media, plus an unknown number through other channels, only 0.08% of participants provided informed consent, indicating significant challenges in recruitment. The A/B testing revealed that the campaign focusing on distanced playful interaction had a significantly higher CTR (1.98%) than the campaign focusing on research participation (CTR=0.51%). In-app activity analysis indicated that 16.4% of participants (n=91) remained active after the first week, but only 5.8% (n=32) stayed active for over 90 days. Interviews highlighted barriers such as excessive chatbot questions and a mismatch between the app’s design and the target group’s preferences. These findings underscore the difficulties in recruitment and retention.

Even though these low participation rates are considered typical in digital epidemiological research [50,51], especially in studies where enrollment is completely web-based [52], evidence for higher participation rates among older adults (though inconclusive) increased expectations [51]. Due to the nature and goal of our app, it is also possible that the stigma on loneliness [15] played a role in the low recruitment rate. Furthermore, the study’s timing during the COVID-19 quarantining period made in-person recruitment strategies (eg, workshops and talks) more challenging, if not impossible.

Most growth came from social media, recruiting more than 200 people in 1 month through Facebook, suggesting participants can be reached. However, few participants completed the entire study. Additionally, when aiming to reach new participants through existing ones, the game did not result in players inviting others once they logged into the app. To that end, improvements to increase game satisfaction must be implemented. This mouth-to-mouth recruitment is considered powerful and should be prioritized [53]. One of the possibilities could be introducing a feature to recommend the game to a Facebook friend and receive in-game rewards. Another way to improve recruitment is to optimize organic user acquisition by, for example, increasing visibility at the top of distributor channel lists like the Apple App Store and Google Play Store, updating metadata often and using metadata keywords based on seasonal contexts [53-55].

After the first week post download, 16.4% of participants remained active; 5.8% remained active after the first 90 days. The questionnaire retention rate remained around 10% compared to the baseline. These retention rates comply with gaming industry standards where a 7-day retention rate of 8.6% and 30-day retention rate of 3.5% in European countries are regular among highly rated gaming apps [56]. Internet-based interventions also experience low retention rates compared to traditional interventions, especially in older populations [57-59]. However, intervention research attrition usually needs to be much lower than that of the gaming industry; attrition of 45% is already described as high [58-61]. Furthermore, player types and participation reasons play a role in retention [62], as differences could exist between players joining for gameplay versus those joining to participate in research. This mismatch between gaming and research standards might imply that initial sample sizes must be much higher than anticipated.

Data collection procedures (ie, backend data collection and chatbot) were considered pleasurable and straightforward. However, language use needs to be adapted per age category as older participants found the chatbot speaking too childlike. The chatbot’s tone is essential as negative emotions regarding the chatbot might affect interaction willingness [63], possibly resulting in communication breakdown and increasing the number of missing values [64]. Furthermore, the questionnaires were considered too long and complicated. However, as the attrition rates after 1 month of play were similar between older adults and the younger group, it seems that questionnaire duration is not the only limiting factor, as the younger group did not need to answer all questionnaires. Additionally, even though interviewees preferred answering questionnaires in the app or on the computer, research shows that most older adults prefer answering questionnaires on paper or, if digitally, on the computer [65]. Therefore, our method of administering questionnaires could have been a barrier.

The games were designed to facilitate inter-generational contact, and participants confirmed they were most suited for children and older adults playing together. However, we found that most people played within their age stratum (ie, good friends and partners), corresponding with the literature suggesting that people of different ages do not uniformly intermingle [66]. This age segregation is not only a matter of preferences [66-69], but also a matter of institutional, spatial, and cultural segregation based on age [67,70-72]. Indicating that the design choices toward intergenerational gameplay did not have their intended effect and possibly even had an adverse impact as older users felt the app was too childish. However, the app is possibly only perceived as “childish” as participants did not interact with it within the context of its intergenerational intention.

### Strengths and Limitations

The first strength of this study is the use of A/B testing as it helped understand recruitment through social media by providing insights into the discrepancy between the extensive reach needed and app downloads realized. Furthermore, it allowed for adjusting recruitment strategies and optimizing recruitment. A second strength is that we provide a detailed qualitative and quantitative evaluation of an innovation built with stakeholders (ie, older persons and game innovation companies), allowing for iterative analysis and improvement in a controlled and verifiable manner. A third strength regards the data collection during the COVID-19 pandemic. Loneliness has increased during COVID-19 [73,74] and many people, especially older adults, had less in-person social contact [75]. Therefore, the need for digital inclusion of older adults increased [76,77]. Furthermore, research shows that older adults use digital technology to improve social connectedness [78,79] and change how they use and adopt new technology during the COVID-19 pandemic [79]. The PlayingTogether app has the potential to fill the need for digital inclusion as it aims to make digital communication easy and accessible, possibly positively influencing some players to engage more with the game.

A first limitation is the timing of data collection during the COVID-19 pandemic as it made in-person recruitment strategies more difficult and therefore reduced inclusion numbers and lessons learned about the effectiveness of in-person recruitment strategies (eg, workshops and presentations). Consequently, recruitment shifted toward digital strategies. Though this made calculating the advertisement reach possible, it possibly caused the substantial filtering of 99.02% which is still a lower estimate as reach increases when adding unknown contributions through different channels. A second limitation is the representativeness of the target group in the in-game sample. Participants were few (ie, N=155), relatively young, highly educated, and not lonely, so the participant pool did not reflect the target group well. By campaigning toward a broad audience, we believed younger players would invite older potential participants; as lonely, more senior adults are hard to reach [80]. Though we successfully recruited a younger target group, this indirect recruitment did not occur, highlighting the difficulty of reaching a diverse group which hampers the game design process and assessment as the target group is underrepresented. A third limitation is that the app was in iterations of active development during the study, including an update on UI. This is also the case in most other eHealth innovations, as the iterations of innovation cannot wait until the end of a (slow) trial evaluation with long-term follow-up. However, making direct comparisons between players became more challenging with several app versions. Nevertheless, the interviewees’ experiences did not differ between versions, indicating that the underlying problems were more profound than those covered by the updates. Finally, our intervention was designed too broad, with the intention to attract both older adults and their grandchildren. We tested game mechanics with older adults and grandchildren, but the intersection of games and questionnaires was not tested. Furthermore, the new UI, although it received the most votes in a digital campaign, was not rigorously tested by older adults and the younger generations. Therefore, the app might have felt “childish” for older adults, and too simple for younger generations, which made the app unsuccessful in connecting the different generations. This might have been overcome by more rigorous pilots and repeated tests within this intended context.

### Future Directions

Based on the data and insights gained in this study, we deem the following matters important to address before further (large-scale) evaluation or implementation of social games in alleviating loneliness in older adults. First, game experiences must provide clear guidance to start and continue gameplay. One option could be to introduce volunteers scouted to keep communication energetic. They could catalyze communication and “break the ice” while monitoring participant activity so necessary changes can promptly be implemented. Second, the intervention needs a sharply defined target group that thoroughly tests the app and the intervention procedures. Our approach of using a mixed target group, comprising both older and younger generations, posed challenges in effectively engaging either demographic. Third, substantial initial recruitment investments and continuous advertisement strategies are essential for long-lasting digital effectiveness trials. Current efforts are insufficient for scaling up, as shown by a 99.92% drop from exposure to download and a 90% dropout rate after 1 month. In-person recruitment could enhance engagement and reduce dropout rates.

There is increasing literature on digital gaming interventions and loneliness [81] and web-based interventions containing a gaming feature and loneliness [82]. However, we believe that further large-scale evaluation only becomes viable once these concerns are addressed. This study adds insights into the gap between the reach of potential participants and the actual recruitment outcome. Furthermore, we have shown how to design, develop, recruit for, and improve games for older adults and where these aspects might need to be improved for future endeavors.

### Conclusions

We describe the development process and participant funnel from advertisement to app usage to provide context and guidance for future work. These stages often remain unreported even when crucial for acceptance and, thus, effectiveness. As such, this study underlines the difficulty of recruiting and retaining older adults in a social gaming intervention focused on loneliness. We showed that combining the target group with a hybrid solution to attract different generations leads to difficulty in providing a user experience where all users understand the app’s intent and feel appropriately addressed. Furthermore, we have shown the discrepancy between advertisement reach and the number of included participants. This filtering process shows that casting a wide net does not necessarily result in high participation rates, indicating that expectations and recruitment strategies should be realistically assessed when initiating such interventions. More focused small-scale, in-person recruitment strategies might therefore be more effective in increasing participation and decreasing attrition.

## Appendix

Multimedia Appendix 1 Sample size calculation.

Multimedia Appendix 2 Participant demographics.

Multimedia Appendix 3 Stages in app design process. An overview of the design process’s different stages (project specification, development, and testing). We first specified a problem statement, followed by identifying the target group. Then a minimal viable product prototype was made and released, and a series of tests and feedback rounds with participants followed.

Multimedia Appendix 4 User interface of the app. Panels A, B, and C show the first user interface in gray tones. Panels D, E, and F show the user interface based on what users found most appealing through a feedback campaign. Panels A and D show the home screen with the participants’ chatgroups. Panels B and E depict the game repository screen with a short explanation of each game and a carousel through which the user can swipe for more games. Panels C and F depict the appearance of a chatgroup, in this case, that of the chatbot administering questionnaires.

Multimedia Appendix 5 Interview guide.

Multimedia Appendix 6 In-depth analysis of participant gameplay.

## Abbreviations

CR: conversion rate
CTR: click-through rate
DJG: De Jong Gierveld Loneliness Scale
DMHI: digital mental health intervention
NDIS: Network Domain Identification and Significance scale
PI: Personal Information Questionnaire
UI: user interface

## Data Availability

The datasets generated and analyzed during this study are not publicly available due to privacy restrictions in accordance with the study sponsor but are available from the corresponding author upon reasonable request.
