# Supplementary material for: Social Gaming to Decrease Loneliness in Older Adults: Recruitment Challenges and Attrition Analysis in a Digital Mixed Methods Feasibility Study
Source: JMIR Serious Games. 2024 Oct 16;12:e52640. doi: 10.2196/52640 (PMC11525082; doi:10.2196/52640)
Supplement: Multimedia Appendix 1 [file games_v12i1e52640_app1.docx]

This study is mainly focused on determining whether social interaction can be measured via gaming. Concurrent validity was assessed by correlating the method employed by the game with the validated NDIS questionnaire [1]. To compute the necessary sample size, we used a commonly applied correlation sample size calculation from [2] using the parameters: α=0.05, β=0.2, and r=0.3. Parameter r, the expected correlation coefficient, is set to 0.3. This is the lowest expected correlation for the hypotheses that will test the construct validity, indicating a weak but statistically significant correlation between social interaction and loneliness.

The calculation yields a total of 85 participants needed for this study. To account for participant dropout, we added 20% on top of the 85 participants required, bringing the total number of participants up to 102. We believe that this number is also feasible from a practical standpoint. The 20% dropout rate is based on a comparable study in a similar population [3].

References

1. van Tilburg TG. Delineation of the social network and differences in network size. Living arrangements and social networks of older adults; 1995;83–96.

2. Hulley, S., Cummings, S., Browner, W., Grady, D., & Newman, T. (2013). Designing clinical research (4th ed.). Philadelphia: LWW.

3. Konstantinidis, E. I., Billis, A. S., Mouzakidis, C. A., Zilidou, V. I., Antoniou, P. E., & Bamidis, P. D. (2014). Design, implementation, and wide pilot deployment of fitforall: an easy to use exergaming platform improving physical fitness and life quality of senior citizens. IEEE Journal of Biomedical and Health Informatics, 20(1), 189–200.
