# Supplementary material for: Social Gaming to Decrease Loneliness in Older Adults: Recruitment Challenges and Attrition Analysis in a Digital Mixed Methods Feasibility Study
Source: JMIR Serious Games. 2024 Oct 16;12:e52640. doi: 10.2196/52640 (PMC11525082; doi:10.2196/52640)
Supplement: Multimedia Appendix 2 [file games_v12i1e52640_app2.docx]

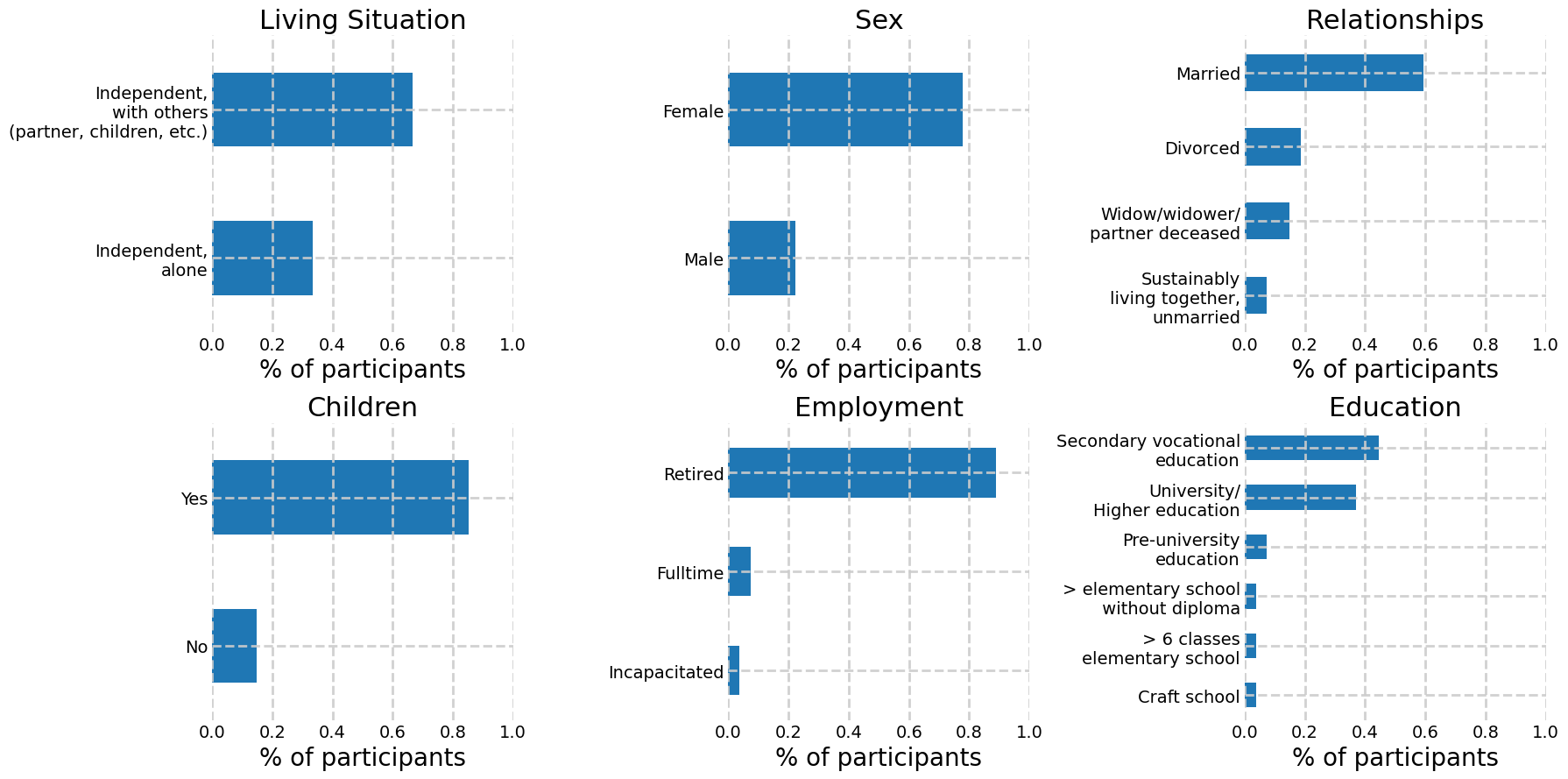


Figure S1: Demographics of participants aged 65 and over. Demographics plots regarding the living situation, sex, relationship status, whether participants have children, employment, and education.


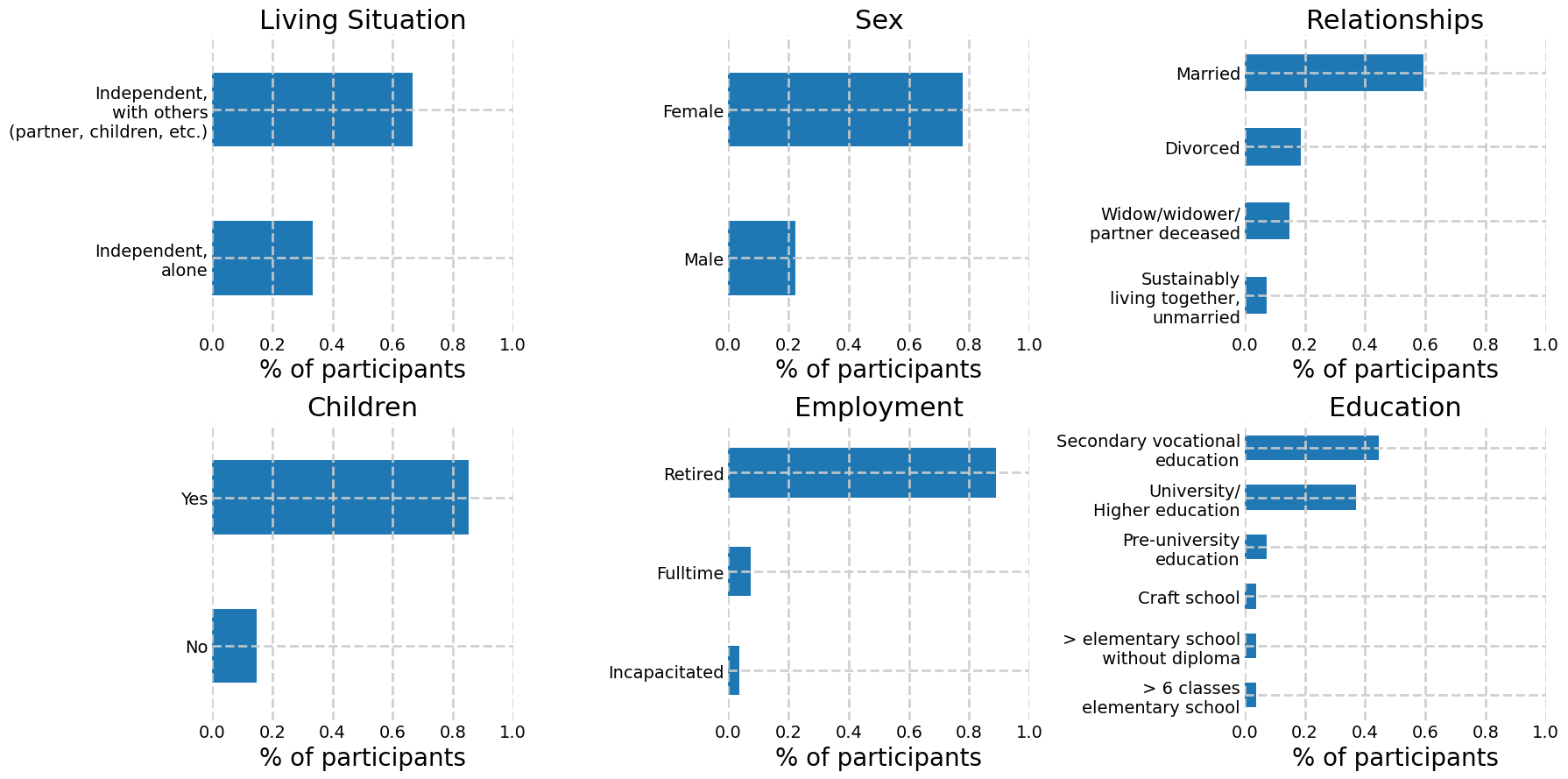


Figure S2: Demographics of participants aged 64 and under.
