# Supplementary figures and images for: Social Gaming to Decrease Loneliness in Older Adults: Recruitment Challenges and Attrition Analysis in a Digital Mixed Methods Feasibility Study
Source: JMIR Serious Games. 2024 Oct 16;12:e52640. doi: 10.2196/52640 (PMC11525082; doi:10.2196/52640)

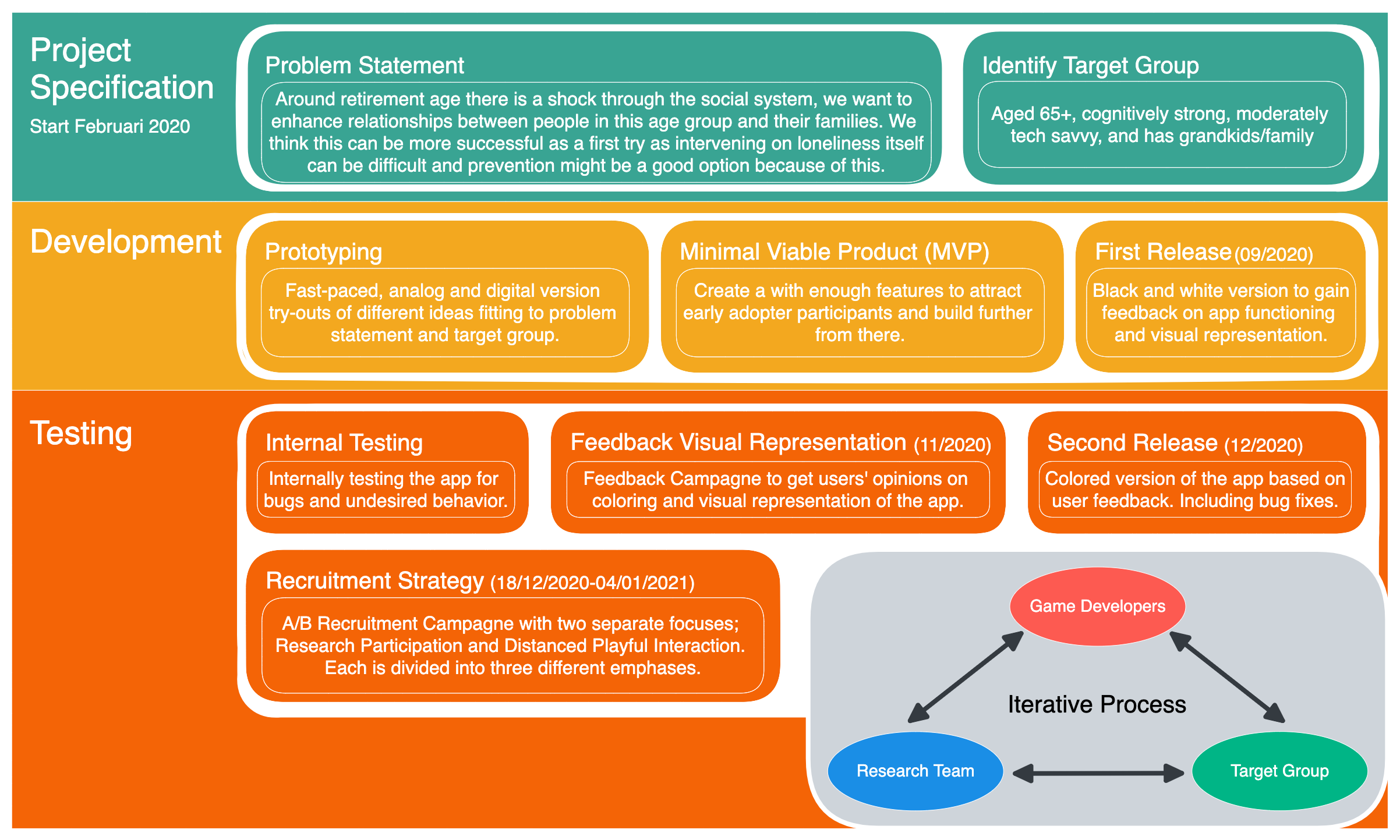

Supplement: Multimedia Appendix 3 [file games_v12i1e52640_app3.png]

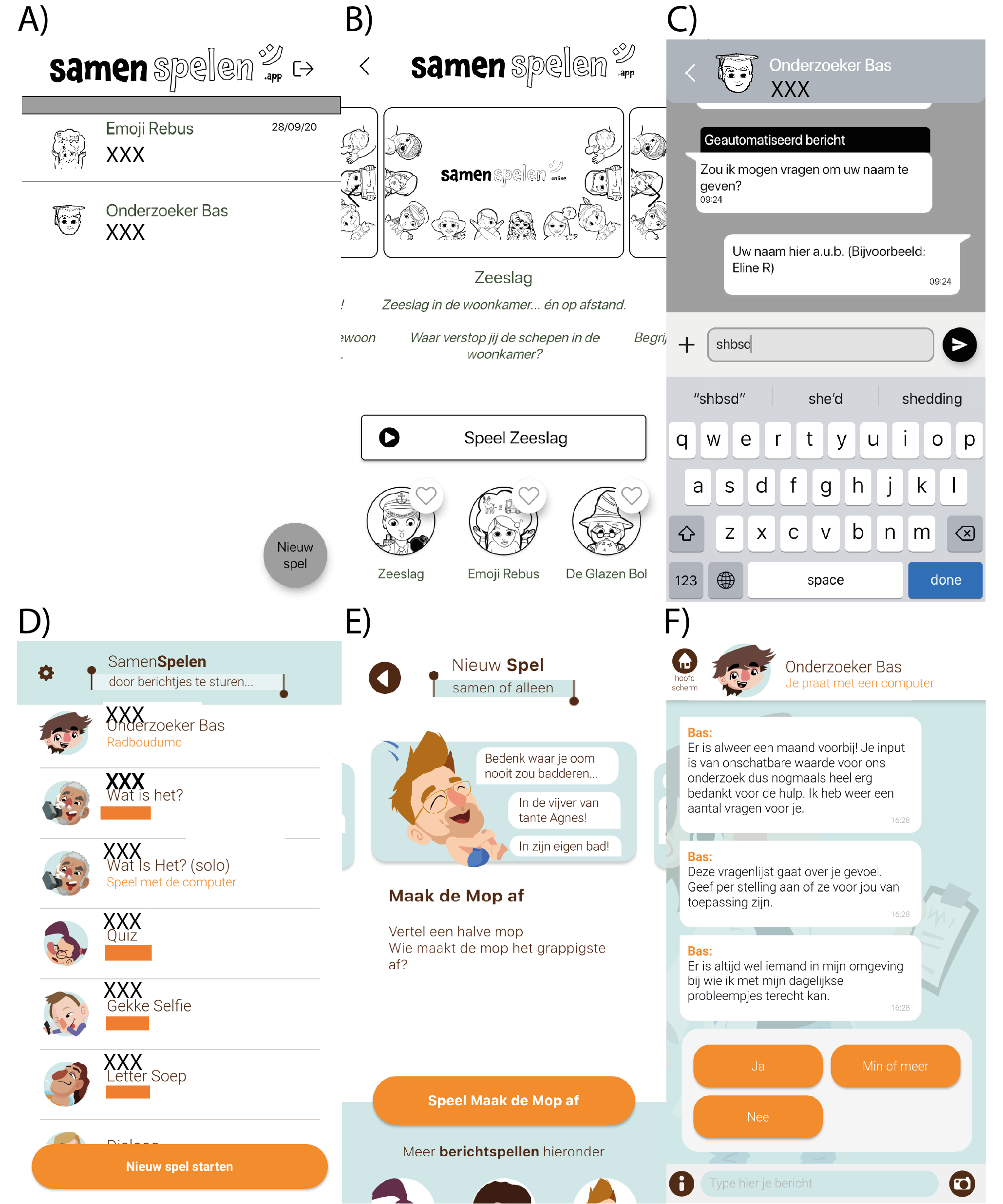

Supplement: Multimedia Appendix 4 [file games_v12i1e52640_app4.png]
