# Supplementary material for: Social Gaming to Decrease Loneliness in Older Adults: Recruitment Challenges and Attrition Analysis in a Digital Mixed Methods Feasibility Study
Source: JMIR Serious Games. 2024 Oct 16;12:e52640. doi: 10.2196/52640 (PMC11525082; doi:10.2196/52640)
Supplement: Multimedia Appendix 5 [file games_v12i1e52640_app5.docx]

### Main questions:

- What is your experience with using the app?
- How often do you like to answer the questionnaires asked by Onderzoeker Bas?
- With whom do you play the most?

### General questions:

- How old are you?
- What is your living situation?
- What is your work situation?
- Do you have children?
  - If yes: How old are they?
- Do you have grandchildren?
  - If yes: How old are they?

### Preliminary phase:

- Why did you download the app?
- How easy or difficult was it to download the app?
  - What made it easy or difficult?
- How easy or difficult was it to start the app?
  - What made it easy or difficult?
- How did you know about the app?

### App usage:

- What is your experience with the app?
- What do you think of the offer of games?
- What do you think of the explanation of the games?
  - Do you immediately know what to do to start playing?
- Did you play a game within the app?
  - If no: why not? What hold you back?
  - If yes: which games? What was your experience? Did you also consider other games?
- How easy or difficult was it to start a game?
  - What made it easy or difficult?
- Did you invite someone else to play a game?
  - Why? Or why not?
- With whom do you play the games the most?
  - Are those people also the people you prefer to play with?
    - If no: why is this different?
- What is already good about the app?
- What can be improved?
- Did you use the old or new version of the app (black and white or colour)?
  - If someone used both versions:
    - What did you think of the app before the update?
    - Have you also used the new version?
    - What do you think of the changes made?
    - What do you think of the new version?

### Onderzoeker Bas:

- What is your experience with Onderzoeker Bas?
- What did you think of the research information?
- What do you think of the questions asked
  - Are the questions clear?
- What do you think of the way of addressing / tone of the questions?
- What do you think of the number of questions asked?
- Do you answer the questions asked by Onderzoeker Bas every time?
  - Why? Or why not?
- One questionnaire is asked more than once, did that already happen to you?
  - If yes: what did you think of the time between the subsequent questionnaires?
- How often do you like to answer the questionnaires asked by Onderzoeker Bas?
- In following research, we want to ask the questions about social contact and perceived loneliness every month (11 questions). What would you think of this?
- Do you prefer answering the questionnaires in the app or on the computer or on paper?
- Do you prefer answering the questionnaires divided over time or right after each other?
