# Supplementary material for: Social Gaming to Decrease Loneliness in Older Adults: Recruitment Challenges and Attrition Analysis in a Digital Mixed Methods Feasibility Study
Source: JMIR Serious Games. 2024 Oct 16;12:e52640. doi: 10.2196/52640 (PMC11525082; doi:10.2196/52640)
Supplement: Multimedia Appendix 6 [file games_v12i1e52640_app6.docx]

#### In-game social networks

Looking at participant gameplay, figure 4A illustrates that 61% of participants did not play with others in the game. The average number of in-game relationships per person aged 65+ was 0.58 (Min = 0, Max = 7, SD = 1.30). On average, participants aged 65- had 1.61 in-game relationships (Min = 0, Max = 20, SD = 2.67).

After filtering out the single-player sessions (Figure 4B), the networks show structures where player communication occurred, referred to as components. Most participants only played with one other person. For components of size three, 12 were not fully connected, meaning that these participants were connected through a single person and therefore did not all play together. There were seven components where one or more players were aged 65+. Three consisted entirely of 65+ participants, three shared a connection with another person aged 65-, and one was the largest component. Figure 4c depicts the largest component consisting of 90 people (1 in the 65+ group, 51 in the 65- group, and 28 of unknown age). Ten research team members were at the core, showing that they had a role in network formation.

Gameplay assessment showed that 67.9% of the 1,363 players did not play with others. The single-person sessions demonstrate the importance of a straightforward invitation process and engaging gameplay. Participants usually formed small, not fully connected, in-game social networks. This lack of closure in the network leaves an opportunity to engage more people and broaden one’s social network by stimulating participants to mix social environments. The largest component contained team members and had several sub-clusters actively playing (both with and without a team member). This suggests that catalysts are needed to guide participants into gameplay and subsequently engage in gameplay amongst themselves. The research team ($N$ = 10) was allowed to play to better understand the app’s functioning, kick-start a more natural word-of-mouth growth, and help where needed; they were not included in further analyses. As this study aimed to assess the app recruitment and inclusion process instead of efficacy, this was deemed acceptable.


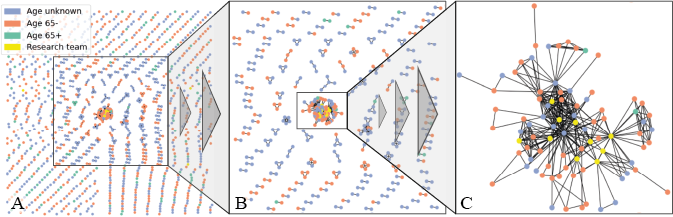


Figure S5: Social networks formed in-game, filtered by component size. Each dot (or node) represents a player, while a line (or edge) between two players represents a relationship. Sending at least one message within the session creates this relationship between two players. *A)* shows all in-game components. *B)* shows all components with size > 1. C) shows the largest component.

#### In-game messaging

In total, 564 players (who at least accepted terms and conditions) sent at least one in-app message. Twenty-two 65+-players sent at least one message with a median of 10 messages ($Q_{1}$ = 2, $Q_{3}$ = 30.75). In the 65- group, 222 players sent at least one in-app message with a median of 6 messages ($Q_{1}$ = 2, $Q_{3}$ = 23.75), and 320 players of unknown age sent a median of 8 messages ($Q_{1}$ = 3, $Q_{3}$ = 28.25).

The median number of sessions participants were part of was 1 ($Q_{1}$ = 1, $Q_{3}$ = 2). Furthermore, the median time between the first and last message was 9 minutes and 20 seconds ($Q_{1}$ = 41.5 seconds, $Q_{3}$ = 3.0 hours). 91 out of 554 players (16.4%) remained active after the first week, and 32 out of the 554 participants (5.8%) had more than 90 days of activity. The app remained available after the study ended; 22 players continued to play. Three participants displayed activity after one year.

### Intervention effectiveness


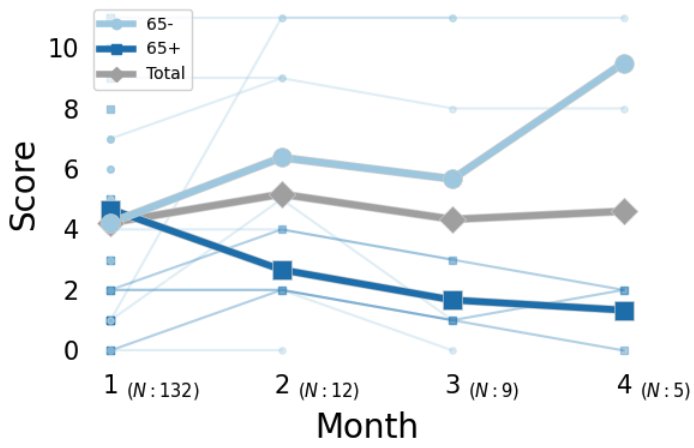
As questionnaire completion rates decreased to 3 out of 29 older participants (~10% of the baseline numbers at the first follow-up) and the data were non-controlled, statistically testing loneliness scores over time was not appropriate. Instead, we depict total loneliness scores over time for all participants in the background. The foreground shows the average loneliness of the 65+ (dark blue squares) and 65- (light blue circles) group and their combined average (gray diamonds). Next to each month on the x-axis, we show the number of participants that provided information for that time. The 65+ group had a mean decrease of 1.67 points in loneliness, caused by a reduction in participants as no 65+ participants displayed a difference between baseline and the last time they answered the questionnaire. The 65- group, on average, increased by 2.64 points in loneliness score over time. In this group, two people had increased loneliness between t0 and their last questionnaire entry (+10 and +1), and two displayed decreased loneliness (-2 and -1). The participant with an increased score of +10 skewed results as the other three participants showed changed scores over time with an average of -0.67 points.

Figure S6: Loneliness over time. Thick lines represent group averages over time, while the thinner lines represent the raw timeseries data of participants.
